# Aridity Modulates N Availability in Arid and Semiarid Mediterranean Grasslands

**DOI:** 10.1371/journal.pone.0059807

**Published:** 2013-04-02

**Authors:** Manuel Delgado-Baquerizo, Fernando T. Maestre, Antonio Gallardo, José L. Quero, Victoria Ochoa, Miguel García-Gómez, Cristina Escolar, Pablo García-Palacios, Miguel Berdugo, Enrique Valencia, Beatriz Gozalo, Zouhaier Noumi, Mchich Derak, Matthew D. Wallenstein

**Affiliations:** 1 Departamento Sistemas Físicos, Químicos y Naturales, Universidad Pablo de Olavide, Sevilla, Spain; 2 Área de Biodiversidad y Conservación, Departamento de Biología y Geología, Escuela Superior de Ciencias Experimentales y Tecnología, Universidad Rey Juan Carlos, Móstoles, Spain; 3 Departamento de Ingeniería Forestal, Campus de Rabanales Universidad de Córdoba, Córdoba, Spain; 4 Natural Resource Ecology Laboratory, Colorado State University, Fort Collins, Colorado, United States of America; 5 Department of Biology, Colorado State University, Fort Collins, Colorado, United States of America; 6 University of Sfax, Département des Sciences de la Vie, Sfax, Tunisia; 7 Direction Régionale des Eaux et Forêts et de la Lutte Contre la Désertification du Rif, Tétouan, Morocco; University of Florida, United States of America

## Abstract

While much is known about the factors that control each component of the terrestrial nitrogen (N) cycle, it is less clear how these factors affect total N availability, the sum of organic and inorganic forms potentially available to microorganisms and plants. This is particularly true for N-poor ecosystems such as drylands, which are highly sensitive to climate change and desertification processes that can lead to the loss of soil nutrients such as N. We evaluated how different climatic, abiotic, plant and nutrient related factors correlate with N availability in semiarid *Stipa tenacissima* grasslands along a broad aridity gradient from Spain to Tunisia. Aridity had the strongest relationship with N availability, suggesting the importance of abiotic controls on the N cycle in drylands. Aridity appeared to modulate the effects of pH, plant cover and organic C (OC) on N availability. Our results suggest that N transformation rates, which are largely driven by variations in soil moisture, are not the direct drivers of N availability in the studied grasslands. Rather, the strong relationship between aridity and N availability could be driven by indirect effects that operate over long time scales (decades to millennia), including both biotic (e.g. plant cover) and abiotic (e.g. soil OC and pH). If these factors are in fact more important than short-term effects of precipitation on N transformation rates, then we might expect to observe a lagged decrease in N availability in response to increasing aridity. Nevertheless, our results suggest that the increase in aridity predicted with ongoing climate change will reduce N availability in the Mediterranean basin, impacting plant nutrient uptake and net primary production in semiarid grasslands throughout this region.

## Introduction

Nitrogen (N) is, after water, the most important factor limiting plant growth, net primary production and microbial decomposition in drylands [1]–[2]. Thus, it is important to develop a predictive understanding of the factors controlling the amount of available N within an ecosystem, which includes both the inorganic (nitrate and ammonium) and organic forms that can be assimilated by plants and microorganisms [3]–[4]. The amount of available N in an ecosystem is controlled by the cumulative effects of microbially-driven N inputs through N-fixation, by the mineralization of N from organic matter, and by N-losses through leaching and gas emissions [5]–[6].

Although temperature and moisture are strong drivers of N transformation rates in soils [7]–[8], the net effect of climate on N availability remains unclear [5]–[6]. For example, N mineralization and decomposition increase with temperature and soil moisture, but high rainfall levels may also enhance denitrification by promoting anaerobic conditions and nitrate leaching [5], [8], [9]–[10]. Some studies have shown that increases in mean annual precipitation enhance the concentration of available N in the field [11]–[12], but others have found the opposite [9]–[10], or have reported inconsistent responses [13]–[14]. Studies focusing on the effects of temperature on N dynamics have also shown inconsistent results [2]. Some experiments show that increases in temperature enhance N availability [8], [15], but others found no significant effects of temperature on the concentration of N in soils [16]. The contradictory results observed to date may be due to differences in plant species, soil types and ecosystems studied, which may determine the effects of temperature and precipitation on soil N availability.

Environmental variables such as plant cover, soil pH, texture and organic matter are also major drivers of N cycling [6], [12], [17]. The uptake of N by plants, as well as the litter and root exudates they produce, affects N concentration in soils [2], [18]. Sandy soils typically exhibit lower denitrification rates, but higher N losses after rainfall events, than clay soils [5], [19]. Plants typically increase the amount of litter mass and soil water moisture under their canopies compared to adjacent bare soil microsites, affecting N mineralization and depolymerization processes [20]–[21]. Acidic soil pH and high organic C concentrations may favor fungal communities, which are involved in depolymerization and decomposition processes, increasing the amount of available N [6], [19], [22]. Finally, the C:N ratio modulates the immobilization and mineralization of N [3], [22].

Despite the emergence of new paradigms for soil N cycling that emphasize the importance of both organic and inorganic N forms [3]–[4], [18], and the growing literature on the topic, we lack an integrated understanding of the most important determinants of N availability. This is particularly true for N-poor ecosystems such as drylands (arid, semiarid and dry-subhumid ecosystems; [22]–[24]). In addition, these areas are more open in terms of their N cycling relative to more humid systems, where most studies have been performed, because they support high N losses relative to N turnover [25]. Drylands are of paramount importance for the Earth system, as they cover about 41% of Earth’s land surface and support over 38% of the total global human population [26]. Improving our knowledge of the factors driving N availability in drylands can greatly enhance our ability to understand how ongoing climate change may affect their functioning [17], and can help to establish “early warning” signals of the onset of desertification processes [26], which often start with the loss of soil nutrients such as N [27].

This study had two main objectives. First, we aimed to evaluate the relationships between N availability (measured as the sum of extractable inorganic and organic N forms) and climatic (aridity), abiotic (soil pH and sand content), plant (average interdistance between plant patches) and nutrient related (organic carbon, N mineralization rate, N transformation rate and the C:N ratio) factors in arid and semiarid grasslands dominated by the tussock grass *Stipa tenacissima* L. along a broad aridity gradient from Spain to Tunisia. They are one of the most widespread and representative ecosystems of the semiarid regions of the Mediterranean basin [28], which is considered highly vulnerable to climate change and desertification [29]. Our second objective was to evaluate whether *S. tenacissima* tussocks modify the relative importance of climate, abiotic, plant and nutrient related factors as potential modulators of N availability. This species is able to create “resource islands” [20], [30]–[31] and modify microclimate, infiltration and soil water availability dynamics [32]–[34] as compared to adjacent bare ground areas. As such, they can potentially alter how climate and other factors affect N availability.

## Methods

### Study Area

This study was carried out in 22 sites located in Spain, Morocco and Tunisia (See map S1), which cover most of the geographic distribution range of *S. tenacissima* grasslands in the Mediterranean basin [28]. All of the sites were located in areas of arid or semiarid climate; total annual precipitation and mean temperature ranged from 141 mm to 465 mm, and from 12.5°C to 20°C, respectively (Table S1). Slope and elevation ranged between 1° and 22°, and between 172 m a.s.l. and 1427 m a.s.l., respectively (Table S1). All sites were located on calcareous soils, and on south-facing slopes. Perennial vegetation cover ranged between 8% and 64%, and was in all cases an open steppe dominated by *S. tenacissima*, with shrub species such as *Quercus coccifera* L., *Rosmarinus officinalis* L. and *Thymus vulgaris* L. in Spain, *Cistus clusii* and *Helianthemum apenninum* L. (Mill.) in Morocco, and *Artemisia herba-alba Asso* and *Hammada scoparia* (Pomel) Iljin in Tunisia.

### Sampling Design and Measurements

At each site, we established a 30 m×30 m plot representative of the dominant vegetation. In the upper left corner of each plot, we located a 30 m transect oriented downslope for the vegetation survey. Three parallel transects of the same length, spaced 8 m apart across the slope, were added. Along each transect, we collected a continuous record of vegetated patches and bare ground areas (i.e. devoid of vascular vegetation). From these transects, we obtained total plant cover, the average interdistance between plant patches and the number of plant patches per 10 m of transect. Vegetation surveys were carried out during 2005 and 2006 (Spain) and in 2010 (Morocco and Tunisia).

We sampled soils in Spain and North Africa (Morocco and Tunisia) during 2006 and 2010, respectively, using a stratified random procedure. Five 50 cm×50 cm quadrats were randomly placed at each of two microsites: bare ground areas and *S. tenacissima* tussocks (Bare and Stipa microsites hereafter). A composite sample consisting of five 145 cm^3^ soil cores (0–7.5 cm depth) was collected from each quadrat, bulked and homogenized in the field. After collection, soils were transported to the laboratory and air-dried at room temperature for four weeks. Previous studies have found that soil biochemical properties are minimally affected by air-drying in semiarid Mediterranean soils [35]. No specific permissions were required for our field activities, except for the site located in Djebel Bou-Hedma Biosphere Reserve and National Park (Tunisia). The required authorization to work at this site was obtained from the Ministère de l’Agriculture, Direction générale des forêts, Tunisia’s authority that manages the Djebel Bou-Hedma Biosphere Reserve and National Park. The study did not involve handling or collection of endangered species.

Organic C (OC) was determined following Anderson and Ingramm [36]. Total N was measured with a CN analyzer (Leco CHN628 Series, Leco Corporation, St Joseph, MI, USA). Total available N was colorimetrically determined from K_2_SO_4_ extracts as the sum of ammonium, nitrate and dissolved organic nitrogen following Delgado-Baquerizo *et al.* [37]. Potential net N transformation (production of available N) and mineralization (production of inorganic-N) rates were measured by determination of the total available and mineral N before and after incubation in the laboratory at 80% of water holding capacity and 30°C for 14 days [23]. Soil pH was measured in all the soil samples with a pH meter, in a 1∶2.5 mass: volume soil and water suspension. One composite sample each per microsite (Bare and Stipa) and plot were analyzed for sand, clay and silt content according to Kettler *et al.* [38].

The UNEP (1992) aridity index (AI = P/PET, where P is annual precipitation and PET is annual potential evapotranspiration) of each site was gathered from the Worldclim global database [39]–[41], as described in Maestre *et al.* [17]. The AI decreases as aridity increases, and to avoid confusing readers we used the inverse of AI in this work. Thus, aridity = 1-AI. This index is strongly related to both annual average rainfall (R^2^ = −0.98) and temperature (R^2^ = 0.80) in our study sites.

### Statistical Analyses

Due to the low number of study sites as compared to number of climatic, abiotic, plant and nutrient variables studied, and the significant correlations between some of the variables from each group (Table S2), we reduced the dimensionality of our dataset by selecting one representative variable for each group (climatic, abiotic, plant and nutrient). We first conducted correlation analyses between the total available N and the different climatic (aridity), abiotic (pH and sand content), plant (total plant and bare cover, average interdistance between plant patches, plant patch area and number of plant patches per 10 m of transect) and nutrient (OC, C:N ratio, potential net mineralization and potential net N transformation rate) variables studied in both Stipa and Bare microsites (Table S3). We then retained for further analyses those variables significantly related to available N in both Stipa and Bare microsites. Aridity, pH and OC were retained as the climatic, abiotic and nutrient variables for subsequent analyses. All of the plant variables were significant related to N availability (Table S3). Thus, we carried out a principal component analysis (PCA) to unify these variables into a single plant component. The first component of this PCA (plant-ax1), which explained the 83.3% of the variance and was the only axis with an eigenvalue higher than 1 (4.167), was retained for further analyses. This component was negatively related to coverage of bare ground and the average plant patch interdistance (r = −0.983 and −0.889, respectively P<0.01 in both cases), but positively related to the *Stipa tenacissima* coverage, plant patch area and number of plant patches per 10 m of transect, respectively (r = 0.932, 0.894 and 0.861, respectively, P<0.01 in all cases).

With this reduced set of independent variables (aridity, pH, OC and plant-ax1), we then used a multi-model inference approach based on information theory and ordinary least squares regression [42] to evaluate their relative importance as drivers of N availability (dependent variable). We ranked all the models that could be generated with our independent variables according to the second-order Akaike information criterion (AIC*_c_*), calculated as described in Fotheringham *et al.* [43]. The AIC*_c_* of each model was then transformed to ΔAIC*_c_*, which is the difference between AIC*_c_* of each model and the minimum AIC*_c_* found for the set of models compared. The ΔAIC*_c_* values were also used to obtain the Akaike weights of each model (w_i_), according to Burnham & Anderson [42]. Akaike’s weights were also used to define the relative importance of each predictor across the full set of models evaluated by summing w_i_ values of all models that include the predictor of interest, taking into account the number of models in which each predictor appears [42]. As we were interested in evaluating whether *S. tenacissima* tussocks modified the importance of the different drivers of N availability evaluated, multi-model analyses were carried out for Bare and Stipa microsites separately. These analyses were conducted with the software SAM 4.0 [44].

**Figure 1:**
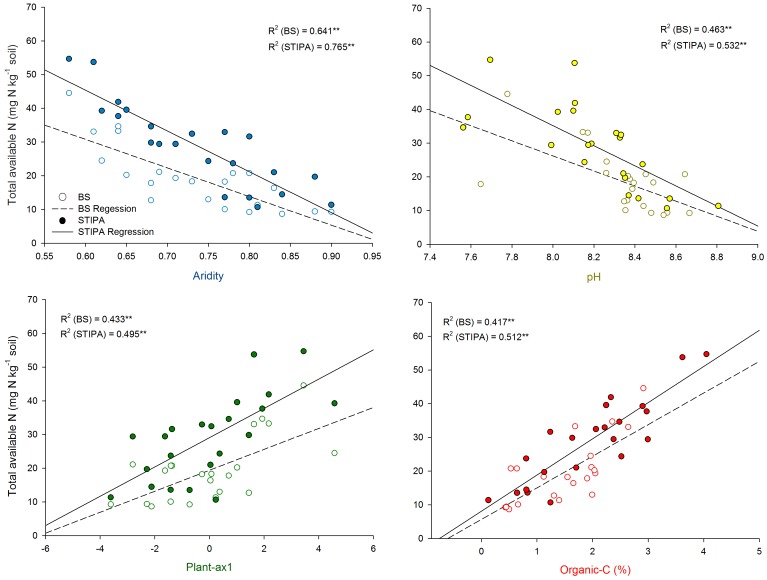
Relationships between total nitrogen (N) availability and aridity, pH, plant-ax1 (first component of a PCA including the cover of bare and plant microsites, average plant patch interdistance, area of plant patches and number of plant patches per 10 m of transect) and organic carbon for both *Stipa tenassicima* (STIPA) and Bare ground (BS) microsites. Every data point is the average of five replicated soil samples. Significance levels are as follows: *p<0.05, **p<0.01 and ***p<0.001.

**Figure 2:**
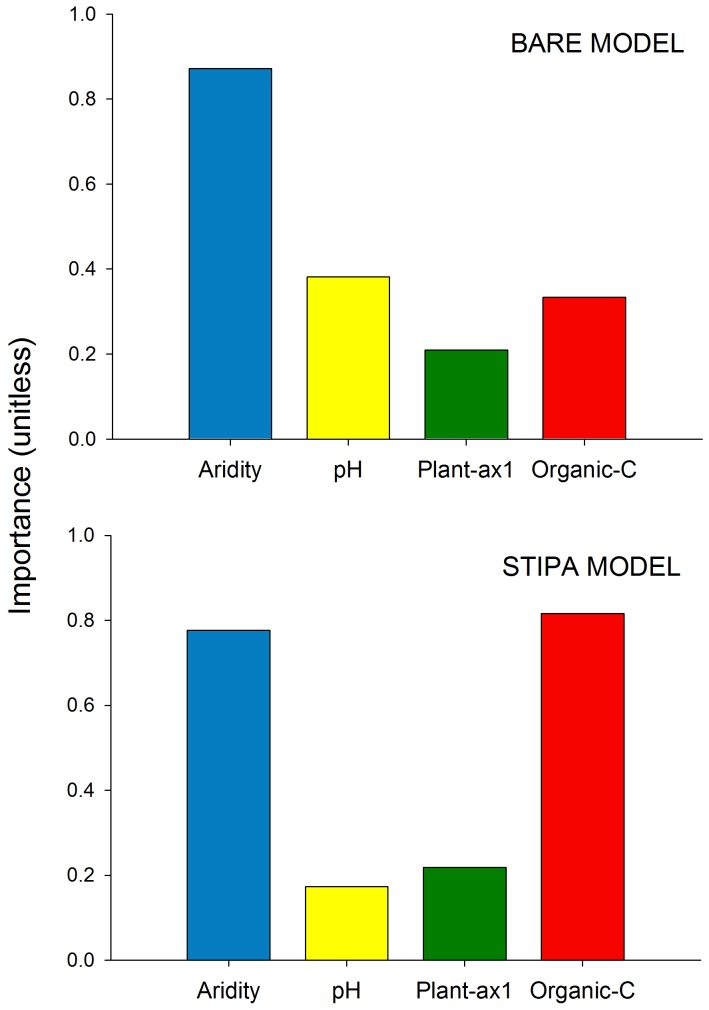
Relative importance of aridity, pH, organic C, and plant-ax1 (first component of a PCA including the cover of bare and plant microsites, average plant patch interdistance, area of plant patches and number of plant patches per 10 m of transect) variables as drivers of variations in N availability. Results are shown for: i) bare ground microsites, and ii) *Stipa tenacissima* microsites. The height of each bar is the sum of the Akaike weights of all models that included the predictor of interest, taking into account the number of models in which each predictor appears.

Aridity and OC, which were highly correlated, had a high explanatory power (Fig. 1) and importance (Fig. 2) among the different predictors of available N that we evaluated (Table S2). Thus, to tease out their relative importance as drivers of N availability, we used structural equation modeling (SEM, [45]). This tool has emerged as a synthesis of path analysis, factor analysis, and maximum likelihood techniques, and has been thoroughly used in the ecological sciences a causal inference tool [46]–[47]. It can test the plausibility of a directed, causal model like that proposed here. Another important capability of SEM is its ability to partition direct and indirect effects that one variable may have on another, and estimate the strengths of these multiple effects. Unlike regression or ANOVA, SEM offers the ability to separate multiple pathways of influence and view them as a system [45]. Thus, the use of SEM is useful for investigating the complex networks of relationships found in natural ecosystems [47]. Path coefficients were obtained using the maximum likelihood estimation technique. These coefficients are interpreted as the size of an effect that one variable exerts upon another. Because of our SEM was saturated (the number of degrees of freedom was zero), no probability level could be assigned to the chi-square statistic, making the model untestable. Thus, the free covariance weight between Aridity and the best solution was chosen through maximization of the maximum likelihood function. The goodness of fit of SEM models was checked following Schermelleh-Engel *et al.* [48]. SEM models were separately conducted for Bare and Stipa microsites with the software AMOS 20 (IBM SPSS Inc, Chicago, IL, USA).

## Results

Soil characteristics changed consistently across the aridity gradient studied. Soil pH increased with increasing aridity for both Stipa and Bare microsites (p<0.01; Table S2). OC decreased with increases in aridity for both microsites (p<0.01; Table S2), but the soil C:N ratio was not related to aridity (p>0.05; Table S2). The cover of *Stipa*, and both the number and area of plant patches were negatively related to aridity (p<0.01; Table S2), while the average interdistance between consecutive plant patches and the cover of bare ground areas increased concomitantly with aridity (p<0.01; Table S2).

Total N availability decreased with aridity for both Stipa (p<0.01) and Bare (p<0.01) microsites (p<0.01; Table S3; Fig. 1). N availability was correlated with both soil and plant characteristics. For example, N availability was positively correlated with pH and OC in both Stipa and Bare microsites (p<0.01;Table S3; Fig. 1). The relationship between N availability and sand content was only significant in the Stipa microsites (p = 0.01; Table S3). We did not find a significant relationship between N availability and the C:N ratio (Table S3). The plant-ax1 was positively related to N availability in both microsites (p<0.01; Table S3; Fig. 1). Total N availability did not appear to be driven by N supply rates, as the relationships between N availability and either potential net mineralization or N transformation rates were non-significant (p>0.05; Table S3).

**Figure 3:**
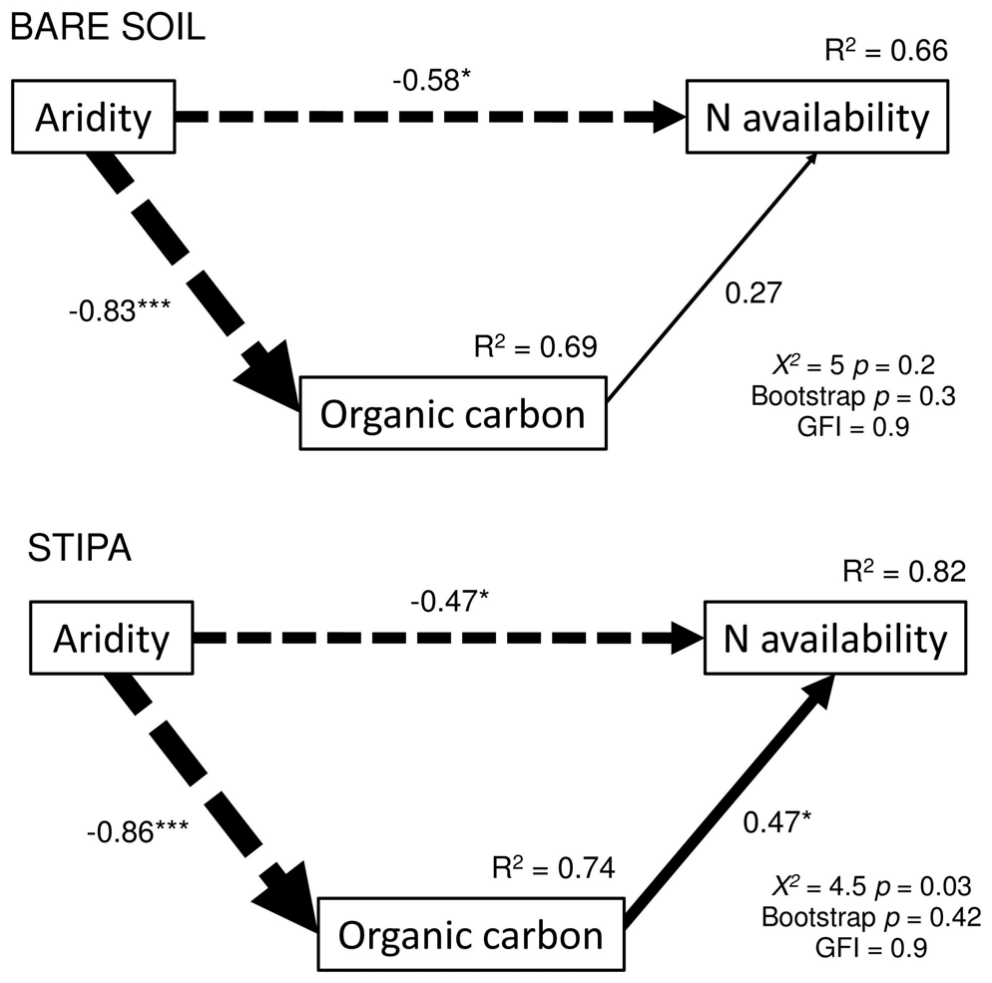
Structural equation models showing the direct and indirect effects of aridity and organic carbon on the total nitrogen availability for the *Stipa tenacissima* (STIPA) and bare ground (BARE SOIL) microsites. Continuous and dashed arrows indicate positive and negative relationships, respectively. Width of arrows is proportional to the strength of path coefficients. The proportion of variance explained (R^2^) appears above every response variable in the model. Goodness-of-fit statistics for each model are shown in the lower right corner. Significance levels are as follows: *p<0.05, **p<0.01 and ***p<0.001.

Multi-model analyses showed that aridity and OC were the most important variables affecting N availability. Aridity explained more of the variability in N availability in Bare microsites (Fig. 2; Table 1), but OC was the most important predictor of N availability in Stipa microsites (Fig. 2; Table 1). The SEMs were satisfactorily fitted to our data, as indicated by goodness-of-fit statistics (Fig. 3; [48]). These analyses revealed that the effects of OC on N availability were largely modulated by aridity, which had larger total effects on N availability than OC in both Bare and Stipa microsites (Fig. 3).

**Table 1:** Top eight best-fitting regression models, ranked according to their AICc value, are presented.

BARE							
Aridity	pH	Plant-ax1	Organic-C	R2	AICc	ΔAIC	Wi
X				0.635	146.24	0	0.31
X	X			0.673	146.89	0.64	0.22
X			X	0.658	147.82	1.58	0.14
X		X		0.638	149.09	2.85	0.07
X	X		X	0.68	149.8	3.55	0.05
X	X	X		0.674	150.17	3.92	0.04
			X	0.561	150.31	4.07	0.03
		X	X	0.605	151.01	4.77	0.04
**STIPA**							
**Aridity**	**pH**	**Plant-ax1**	**Organic-C**	**R2**	**AICc**	**ΔAIC**	**Wi**
X			X	0.82	145.83	0	0.41
X				0.77	148.32	2.49	0.12
			X	0.76	148.51	2.68	0.11
X	X		X	0.82	149.03	3.2	0.08
X		X	X	0.82	149.09	3.27	0.08
		X	X	0.79	149.2	3.37	0.08
X	X			0.77	151	5.17	0.03
X		X		0.77	151.28	5.45	0.01

AICc measures the relative goodness of fit of a given model; the lower its value, the more likely the model to be correct. Aridity, pH, plant-ax1 and organic C were included in these models. Bare = data from bare ground soils only, and Stipa = data from *Stipa tenacissima* soils only.

## Discussion

Evaluating which abiotic and biotic factors drive the N cycle has been an important research focus over the last two decades. It has been motivated by the importance of N for the functioning of the Earth system, and by anthropogenic activities affecting N cycling in a range of ecosystems [42]. Yet, most of the studies carried out on this topic have focused on the effects of single variables, and relatively few of them have been carried out in drylands [11], [13]–[14].

Our results indicate that aridity is the most important driver of N availability in the studied grasslands, highlighting the importance of abiotic controls of the N cycle in drylands. This availability was strongly correlated with multiple soil and plant variables (Table S2), which are also highly related to different aspects of the N cycle [5]–[6]. Aridity also appears to be a strong driver of OC, which was strongly correlated with N availability in this study. Increases in OC may enhance the activity of heterotrophic fungi and bacterial communities that carry out depolymerization and mineralization processes, increasing N availability [22].

Plant and soil properties were also correlated with N availability. Reductions in aridity along the environmental gradient were associated with increases in the plant-ax1, i.e. in the cover, area and number of *S. tenacissima* patches (Table S2). As plant cover increases across the aridity gradient, N and C inputs to the soil from litter are also likely to increase, as indicated by higher decomposition rates under *S. tenacissima* canopies than on surrounding bare ground areas [49]–[50]. The link between plant cover and N availability is likely to be most evident in ecosystems with low organic matter content, such as those studied, where existing substrate pools in the soil are small relative to the inputs of nutrients entering the soil from plant detritus [51]. Increases in rainfall (lower aridity conditions) may favor N leaching and soil formation processes, decreasing the pH and sand content of the soil, which may increase the retention of OC and favor fungal communities involved in N mineralization and depolymerization processes [5]–[6]. We observed an increase in N availability with decreases in soil pH and increases in OC contents, consistent with previous studies [6], [22]. However, the relationship between N availability and OC in drylands is not straightforward because N availability should be more related to microbial biomass turnover and atmospheric inputs than to soil organic matter conent [52]–[54].

Other factors, such as potential net N mineralization and transformation rates, and the C:N ratio, were poorly related to N availability. These results were unexpected, as N transformation rates (which include depolymerization and N mineralization) should be strongly linked to N availability [18]. Our results suggest that potential net N transformation rates, conducted under laboratory conditions (30°C and 80% of water holding capacity), may not be a good indicator of soil N availability in arid and semiarid ecosystems because they do not take into account the effects of drying-rewetting cycles on microbial and plant processes, including rapid microbial turnover and plant nutrient uptake and losses through leaching or gaseous emissions [23], [55]. A decrease in the C:N ratio has been suggested to increase N mineralization [22]. However, this ratio did not show a significant relationship with the amount of available N in this study. This finding suggests that the C:N ratio may not be a good indicator of N availability in this system, possibly because this ratio includes both labile and recalcitrant C, the latter being less available for microorganisms [2], [22].

Important differences were found among microsites when modeling N availability. While aridity was the most important driver of changes in N availability in the Bare model, OC was as important as aridity in Stipa microsites. Soils at this microsite had more OC than soils in Bare microsites in most of the sites surveyed (14 out of 22 sites, Table S4). Similar results have been found in other *S. tenacissima* steppes (e.g. [20]). Increasing OC content may drive increased biomass of heterotrophic microbial communities (fungi and bacteria), which drive N mineralization and depolymerization processes, increasing N availability and favoring a fertility island effect under the canopy of *S. tenacissima* [2], [6], [20], [30]–[34]. The effect of *S. tenacissima* on microclimate and soils beneath their canopies, including lower temperature and higher moisture compared to bare ground areas [20], [33], may have also promoted N mineralization and depolymerization processes.

Studies aiming to evaluate how N availability changes along regional climatic gradients have been carried out in drylands from the USA [14], Argentina [12] and South Africa [11]. However, none of them considered both organic and inorganic N availability. Our results indicate that aridity is the most important factor modulating total N availability in Mediterranean *S. tenacissima* grasslands. In addition, four different organic (DON and amino acids) and inorganic (ammonium and nitrate) N forms were positively related to N availability but negatively related to aridity, showing that decreasing N availability with increasing aridity may affect both organic and inorganic N forms available to plants and microorganisms (Figure S1 and S2). This suggests that changes in precipitation regimes are likely to affect N availability, albeit the temporal scale of this response is uncertain. Climate change models predict reductions in rainfall and increases in temperature throughout the semiarid and arid areas of the Mediterranean basin [29]. While soil moisture is a proximate driver of N transformation rates, our results suggest that N transformation rates are not the most important drivers of N availability. Rather, the strong relationship between aridity and N availability could be driven by indirect effects that operate over longer time scales (decades to millennia) including both biotic (e.g. plant cover) and abiotic (e.g. soil OC and pH). If these factors are in fact more important than short-term effects of precipitation on N transformation rates, then we might expect to observe a lagged decrease in N availability in response to increasing aridity. If N availability does decrease in response to climate change, it will have important harmful effects on plant nutrition and net primary production within *S. tenacissima* grasslands, and will also negatively impact other key supporting (e.g. soil conservation, [50]), provisioning (grazing, [56]) and cultural (e.g. game hunting, [57]) ecosystem services provided by this ecosystem throughout the Mediterranean basin.

## Supporting Information

Figure S1 **Pearsońs relationships between organic (DON and amino acids) and inorganic (ammonium and nitrate) N forms with aridity for both** ***Stipa tenassicima*** **(STIPA) and Bare soil (BS) microsites.** Every data point is the average of five soil samples. Significance levels are as follows: *p<0.05, **p<0.01 and ***p<0.001. Ammonium, nitrate and DON were measured as described by Delgado-Baquerizo et al. (1). Amino acids were determined by following Chantigny et al. (2).
(DOC)

Figure S2 **Relationships between organic (DON and amino acids) and inorganic (ammonium and nitrate) N forms with total available N for both Stipa tenassicima (STIPA) and Bare soil (BS) microsites.** Every data point is the average of five soil samples. Significance levels are as follows: *p<0.05, **p<0.01 and ***p<0.001. Ammonium, nitrate and DON were measured as described by Delgado-Baquerizo et al. (1). Amino acids were determined by following Chantigny et al. (2).
(DOC)

Table S1 **Location, climatic, physical and main soil chemical characteristics in the studied sites.** MAT = Mean annual temperature; MAP = Mean annual precipitation; Stipa = coverage of Stipa.
(DOC)

Table S2 **Pearson correlations coefficients.** between the different climatic (aridity), abiotic (pH; SAC: % of sand content), plant (CBA: % of coverage of bare ground; CHE: % of coverage of *Stipa tenacissima*; PA: plant patch area [m^2^]; API: Average plant patch interdistance [m]; NP: number of plant patches per 10 m of transect) and nutrient (Organic-C [%]; MIN; potential net mineralization rate [mg N kg^−1^ soil day^−1^]; NRT: potential net N transformation rate [mg N kg^−1^ soil day^−1^]; ratio C:N) variables. Significance levels are as follows: *p<0.05 and **and p<0.01.
(DOC)

Table S3 **Pearson correlations coefficients.** between available N and climatic (aridity), abiotic (pH; SAC: sand content), plant (CBA: coverage of bare ground; CHE: coverage of *Stipa tenacissima*; PA: plant patch area; API: Average plant patch interdistance; NP: number of plant patches per 10 m of transect) and nutrient (Organic-C; MIN; potential net mineralization rate; NRT: potential net N transformation rate; ratio C:N) variables. Significance levels are as follows: *p<0.05 and **and p<0.01.
(DOC)

Table S4 **Summary results of the semi-parametric PERMANOVA analyses carried out with organic carbon.** PERMANOVA uses permutation tests to obtain p values, does not rely on the assumptions of traditional parametric ANOVA, and can handle experimental designs such as employed here (1). The model used evaluated the effects of plot (PL as random factor) and microsite (MI as fixed factor) on organic carbon.When significant interactions between factors were found, separate PERMANOVA analysis were conducted for each site. All analyses were carried out using the PERMANOVA+ module of the PRIMER software (PRIMER-E Ltd., Plymounth Marine Laboratory, UK). RES = residuals.
(DOC)
