# Supplementary material for: Aridity Modulates N Availability in Arid and Semiarid Mediterranean Grasslands
Source: PLoS One. 2013 Apr 2;8(4):e59807. doi: 10.1371/journal.pone.0059807 (PMC3614980; doi:10.1371/journal.pone.0059807)
Supplement: Figure S2 — Relationships between organic (DON and amino acids) and inorganic (ammonium and nitrate) N forms with total available N for both Stipa tenassicima (STIPA) and Bare soil (BS) microsites. Every data point is the average of five soil samples. Significance levels are as follows: *p<0.05, **p<0.01 and ***p<0.001. Ammonium, nitrate and DON were measured as described by Delgado-Baquerizo et al. (1). Amino acids were determined by following Chantigny et al. (2). (DOC) [file pone.0059807.s002.doc]

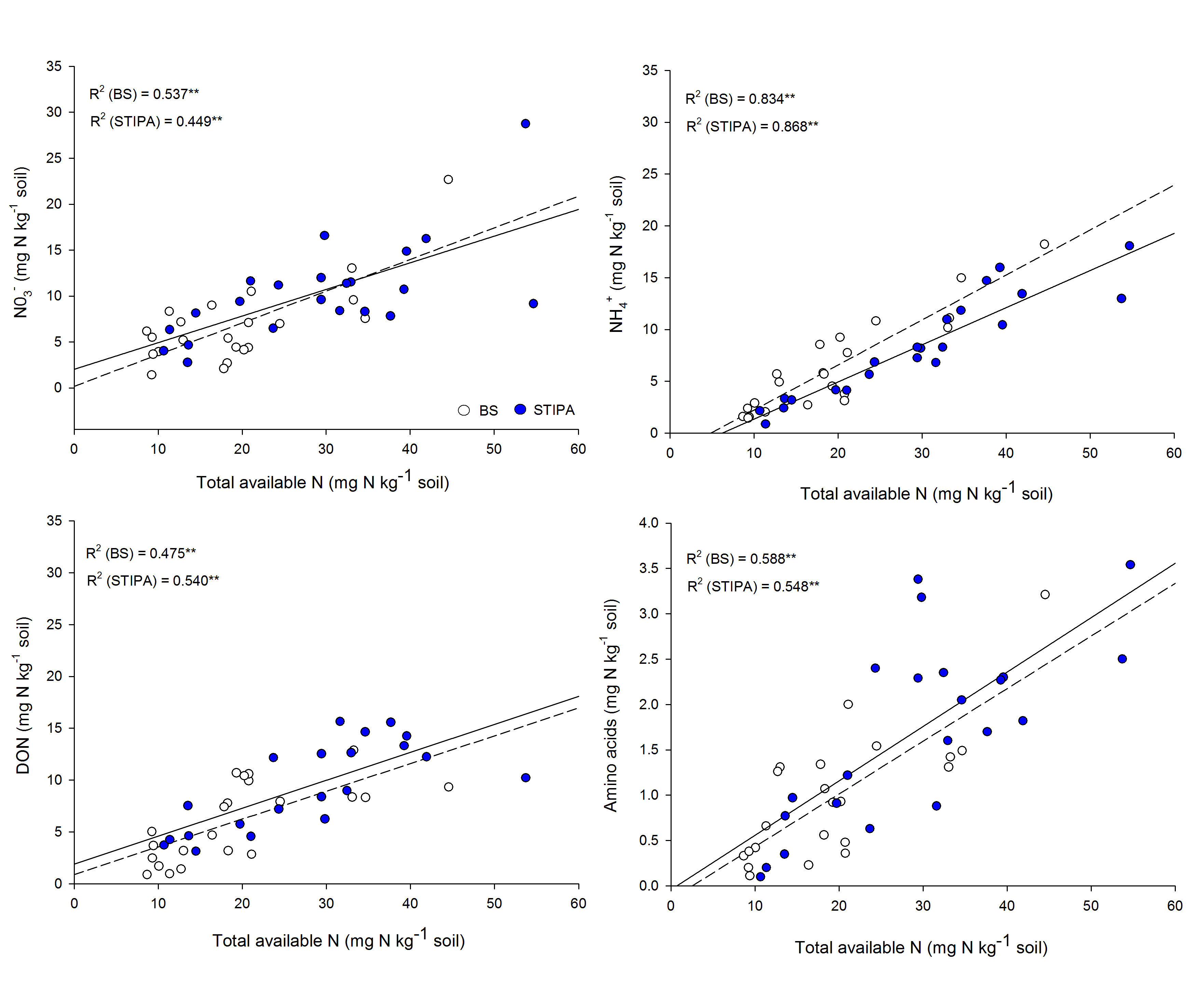


**Figure S2.** Relationships between organic (DON and amino acids) and inorganic (ammonium and nitrate) N forms with total available N for both Stipa tenassicima (STIPA) and Bare soil (BS) microsites. Every data point is the average of five soil samples. Significance levels are as follows: * p < 0.05,** p < 0.01 and *** p < 0.001. Ammonium, nitrate and DON were measured as described by Delgado-Baquerizo et al. (1). Amino acids were determined by following Chantigny et al. (2).

References:

1. Delgado-Baquerizo M, Castillo-Monroy AP, Maestre FT, Gallardo A (2010) Change in the dominance of N forms within a semi-arid ecosystem. Soil Biology Biochemistry 42: 376– 378.

2. Chantigny MH, Angers DA, Kaiser K, Kalbitz K (2006). Extraction and Characterization of Dissolved Organic Matter. In: Carter, M.R, Gregorich, E.G., Eds., Soil Sampling and Methods of analysis, Canadian soil of society science.
