# Supplementary material for: Aridity Modulates N Availability in Arid and Semiarid Mediterranean Grasslands
Source: PLoS One. 2013 Apr 2;8(4):e59807. doi: 10.1371/journal.pone.0059807 (PMC3614980; doi:10.1371/journal.pone.0059807)
Supplement: Table S1 — Location, climatic, physical and main soil chemical characteristics in the studied sites. MAT = Mean annual temperature; MAP = Mean annual precipitation; Stipa = coverage of Stipa. (DOC) [file pone.0059807.s003.doc]

**Table S1.** Location, climatic, physical and main soil chemical characteristics in the studied sites. MAT = Mean annual temperature; MAP = Mean annual precipitation; Stipa= coverage of Stipa.

| Site name | Country | Elevation (m) | Lattitude | Longitude | MAT (ºC) | MAP (mm) | Slope (º) | Sand (%) | Clay (%) | Silt (%) | pH (H_2_0) | Stipa (%) |
| --- | --- | --- | --- | --- | --- | --- | --- | --- | --- | --- | --- | --- |
| Frissate | Morocco | 1349 | 33°03' N | 2°25 W | 15.3 | 283 | 1.75 | 81.59 | 3.56 | 14.85 | 8.53 | 28.33 |
| Guenfouda | Morocco | 956 | 34°25' N | 2°11' W | 14.5 | 321 | 5.75 | 52.89 | 5.92 | 41.19 | 8.27 | 36.71 |
| Guercif | Morocco | 782 | 33°58' N | 3°22' W | 16.0 | 265 | 18.25 | 56.87 | 7.54 | 35.59 | 8.34 | 16.23 |
| Mezguitem2 | Morocco | 982 | 34°26' N | 3°35' W | 14.8 | 399 | 14.25 | 54.25 | 4.80 | 40.95 | 8.28 | 45.67 |
| Ogda | Morocco | 1155 | 34°18' N | 1°59' W | 13.9 | 377 | 7.00 | 51.51 | 4.80 | 43.69 | 8.36 | 17.30 |
| Sabbab1 | Morocco | 1013 | 33°52' N | 3°38' W | 14.9 | 307 | 7.25 | 66.28 | 4.45 | 29.27 | 8.37 | 30.04 |
| Sabbab2 | Morocco | 753 | 33°55' N | 3°33' W | 16.3 | 289 | 10.75 | 72.62 | 5.12 | 22.26 | 8.49 | 25.96 |
| Sahibat | Morocco | 1430 | 33°04' N | 2°43' W | 14.6 | 310 | 5.75 | 67.52 | 3.14 | 29.34 | 8.37 | 16.96 |
| Saka1 | Morocco | 733 | 34°37' N | 3°24' W | 15.9 | 339 | 20.00 | 55.55 | 6.69 | 37.76 | 8.46 | 28.13 |
| Saka2 | Morocco | 936 | 34°37' N | 3°27' W | 14.7 | 385 | 16.50 | 42.78 | 16.21 | 41.00 | 8.31 | 7.96 |
| Barrax | Spain | 785 | 39°02' N | 2°13' W | 13.7 | 415 | 4.50 | 45.53 | 21.16 | 33.31 | 8.24 | 45.58 |
| Huelves | Spain | 891 | 40°04' N | 2°54' W | 12.5 | 465 | 21.50 | 48.76 | 6.80 | 44.44 | 7.72 | 59.58 |
| Morata | Spain | 627 | 40°12' N | 3°25' W | 14.0 | 432 | 22.00 | 54.11 | 6.30 | 39.59 | 8.08 | 63.88 |
| Ontígola | Spain | 593 | 39°59' N | 3°37' W | 14.5 | 412 | 10.25 | 47.64 | 4.49 | 47.87 | 8.10 | 47.17 |
| Sierra Espuña | Spain | 663 | 37°49' N | 1°40' W | 15.1 | 378 | 1.25 | 49.95 | 5.77 | 44.28 | 7.61 | 38.50 |
| Yecla | Spain | 819 | 38°35' N | 1°11' W | 13.9 | 444 | 18.50 | 61.14 | 6.14 | 32.72 | 8.14 | 29.25 |
| Zorita | Spain | 632 | 40°21' N | 2°52' W | 13.6 | 405 | 8.00 | 72.69 | 9.28 | 18.03 | 7.59 | 31.33 |
| Bouhedma | Tunisia | 239 | 34º29' N | 9º38' E | 18.2 | 177 | 4.00 | 62.09 | 5.65 | 32.26 | 8.51 | 9.96 |
| Matmata | Tunisia | 546 | 33º31' N | 9º58' E | 18.7 | 221 | 22.00 | 65.87 | 5.75 | 28.38 | 8.48 | 10.50 |
| Sbeitla | Tunisia | 674 | 35º09' N | 9º06' E | 17.1 | 274 | 4.00 | 59.81 | 5.46 | 34.74 | 8.49 | 41.83 |
| Sidi Bousid | Tunisia | 523 | 34º57' N | 9º43' E | 17.4 | 233 | 18.00 | 58.93 | 3.85 | 37.22 | 8.37 | 34.92 |
| Tataouine | Tunisia | 303 | 32º59' N | 10º29' E | 20.0 | 141 | 1.00 | 81.17 | 5.33 | 13.51 | 8.50 | 7.50 |
