# Supplementary material for: Aridity Modulates N Availability in Arid and Semiarid Mediterranean Grasslands
Source: PLoS One. 2013 Apr 2;8(4):e59807. doi: 10.1371/journal.pone.0059807 (PMC3614980; doi:10.1371/journal.pone.0059807)
Supplement: Table S2 — Pearson correlations coefficients. between the different climatic (aridity), abiotic (pH; SAC: % of sand content), plant (CBA: % of coverage of bare ground; CHE: % of coverage of Stipa tenacissima; PA: plant patch area [m2]; API: Average plant patch interdistance [m]; NP: number of plant patches per 10 m of transect) and nutrient (Organic-C [%]; MIN; potential net mineralization rate [mg N kg−1 soil day−1]; NRT: potential net N transformation rate [mg N kg−1 soil day−1]; ratio C:N) variables. Significance levels are as follows: *p<0.05 and **and p<0.01. (DOC) [file pone.0059807.s004.doc]

**Table S2.** Pearson correlations coefficients between the different climatic (aridity), abiotic (pH; SAC: % of sand content), plant (CBA: % of coverage of bare ground; CHE: % of coverage of *Stipa tenacissima*; PA: plant patch area [m^2^]; API: Average plant patch interdistance [m]; NP: number of plant patches per 10 m of transect) and nutrient (Organic-C [%]; MIN; potential net mineralization rate [mg N kg^-1^ soil day^-1^]; NRT: potential net N transformation rate [mg N kg^-1^ soil day^-1^]; ratio C:N) variables. Significance levels are as follows: * p < 0.05 and ** and p < 0.01.

| **BARE** | **Aridity** | **SAC** | **pH** | **CBA** | **CHE** | **PA** | **API** | **NP** | **Organic-C** | **MIN** | **NTR** | **C:N** |
| --- | --- | --- | --- | --- | --- | --- | --- | --- | --- | --- | --- | --- |
| **Aridity** | 1 |  |  |  |  |  |  |  |  |  |  |  |
| **SAC** | 0.588** | 1 |  |  |  |  |  |  |  |  |  |  |
| **pH** | 0.676** | 0.298 | 1 |  |  |  |  |  |  |  |  |  |
| **CBA** | 0.732** | 0.297 | 0.535* | 1 |  |  |  |  |  |  |  |  |
| **CHE** | -0.657** | -0.427* | -0.412 | -0.916** | 1 |  |  |  |  |  |  |  |
| **PA** | -0.669** | -0.336 | -0.394 | -0.923** | 0.873** | 1 |  |  |  |  |  |  |
| **API** | 0.723** | 0.385 | 0.554** | 0.835** | -0.745** | -0.661** | 1 |  |  |  |  |  |
| **NP** | -0.813** | -0.287 | -0.663** | -0.806** | 0.705** | 0.608** | -0.825** | 1 |  |  |  |  |
| **Organic-C** | -0.835** | -0.586** | -0.719** | -0.585** | 0.575** | 0.472* | -0.623** | 0.830** | 1 |  |  |  |
| **MIN** | -0.647** | -0.704** | -0.419 | -0.485* | 0.537** | 0.555** | -0.433* | 0.548** | 0.723** | 1 |  |  |
| **NTR** | -0.591** | -0.585** | -0.368 | -0.481* | 0.527* | 0.583** | -0.382 | 0.419 | 0.609** | 0.682** | 1 |  |
| **C:N** | 0.369 | 0.066 | 0.516* | 0.092 | -0.042 | -0.02 | 0.023 | -0.442* | -0.614** | -0.377 | -0.255 | 1 |
|  |  |  |  |  |  |  |  |  |  |  |  |  |
| **STIPA** |  |  |  |  |  |  |  |  |  |  |  |  |
| **Aridity** | 1 |  |  |  |  |  |  |  |  |  |  |  |
| **SAC** | 0.566** | 1 |  |  |  |  |  |  |  |  |  |  |
| **pH** | 0.792** | 0.545** | 1 |  |  |  |  |  |  |  |  |  |
| **CBA** | 0.732** | 0.277 | 0.564** | 1 |  |  |  |  |  |  |  |  |
| **CHE** | -0.657** | -0.399 | -0.456* | -0.916** | 1 |  |  |  |  |  |  |  |
| **PA** | -0.669** | -0.306 | -0.450* | -0.923** | 0.873** | 1 |  |  |  |  |  |  |
| **API** | 0.723** | 0.406 | 0.628** | 0.835** | -0.745** | -0.661** | 1 |  |  |  |  |  |
| **NP** | -0.813** | -0.309 | -0.662** | -0.806** | 0.705** | 0.608** | -0.825** | 1 |  |  |  |  |
| **Organic-C** | -0.870** | -0.628** | -0.817** | -0.607** | 0.526* | 0.494* | -0.656** | 0.816** | 1 |  |  |  |
| **MIN** | -0.219 | -0.592** | -0.371 | 0.087 | -0.053 | -0.184 | -0.138 | 0.211 | 0.512* | 1 |  |  |
| **NTR** | -0.249 | -0.482* | -0.261 | 0.095 | -0.088 | -0.217 | -0.149 | 0.28 | 0.484* | 0.868** | 1 |  |
