# Supplementary material for: Aridity Modulates N Availability in Arid and Semiarid Mediterranean Grasslands
Source: PLoS One. 2013 Apr 2;8(4):e59807. doi: 10.1371/journal.pone.0059807 (PMC3614980; doi:10.1371/journal.pone.0059807)
Supplement: Table S3 — Pearson correlations coefficients. between available N and climatic (aridity), abiotic (pH; SAC: sand content), plant (CBA: coverage of bare ground; CHE: coverage of Stipa tenacissima; PA: plant patch area; API: Average plant patch interdistance; NP: number of plant patches per 10 m of transect) and nutrient (Organic-C; MIN; potential net mineralization rate; NRT: potential net N transformation rate; ratio C:N) variables. Significance levels are as follows: *p<0.05 and **and p<0.01. (DOC) [file pone.0059807.s005.doc]

**Table S3.** Pearson correlations coefficients between available N and climatic (aridity), abiotic (pH; SAC: sand content), plant (CBA: coverage of bare ground; CHE: coverage of *Stipa tenacissima*; PA: plant patch area; API: Average plant patch interdistance; NP: number of plant patches per 10 m of transect) and nutrient (Organic-C; MIN; potential net mineralization rate; NRT: potential net N transformation rate; ratio C:N) variables. Significance levels are as follows: * p < 0.05 and ** and p < 0.01.

|  | **BARE** | **STIPA** |
| --- | --- | --- |
|  | **Available N** | **Available N** |
| **Available N (mg N kg^-1^ soil)** | 1 | 1 |
| **Aridity** | -0.797** | -0.875** |
| **Sand content (%)** | -0.357 | -0.538** |
| **pH (H_2_0)** | -0.681** | -0.730** |
| **CBA (%)** | -0.621** | -0.651** |
| **CHE (%)** | 0.500* | 0.546** |
| **PA (m^2^)** | 0.508* | 0.544** |
| **API (m)** | -0.646** | -0.716** |
| **NP** | 0.743** | 0.769** |
| **Organic-C (%)** | 0.749** | 0.874** |
| **C:N ratio** | -0.381 | -0.31 |
| **MIN (mg N kg^-1^ soil day^-1^)** | 0.41 | 0.293 |
| **NTR (mg N kg^-1^ soil day^-1^)** | 0.193 | 0.266 |
