# Supplementary material for: Aridity Modulates N Availability in Arid and Semiarid Mediterranean Grasslands
Source: PLoS One. 2013 Apr 2;8(4):e59807. doi: 10.1371/journal.pone.0059807 (PMC3614980; doi:10.1371/journal.pone.0059807)
Supplement: Table S4 — Summary results of the semi-parametric PERMANOVA analyses carried out with organic carbon. PERMANOVA uses permutation tests to obtain p values, does not rely on the assumptions of traditional parametric ANOVA, and can handle experimental designs such as employed here (1). The model used evaluated the effects of plot (PL as random factor) and microsite (MI as fixed factor) on organic carbon.When significant interactions between factors were found, separate PERMANOVA analysis were conducted for each site. All analyses were carried out using the PERMANOVA+ module of the PRIMER software (PRIMER-E Ltd., Plymounth Marine Laboratory, UK). RES = residuals. (DOC) [file pone.0059807.s006.doc]

**Table S4.** Summary results of the semi-parametric PERMANOVA analyses carried out with organic carbon. PERMANOVA uses permutation tests to obtain p values, does not rely on the assumptions of traditional parametric ANOVA, and can handle experimental designs such as employed here (1). The model used evaluated the effects of plot (PL as random factor) and microsite (MI as fixed factor) on organic carbon.When significant interactions between factors were found, separate PERMANOVA analysis were conducted for each site. All analyses were carried out using the PERMANOVA+ module of the PRIMER software (PRIMER-E Ltd., Plymounth Marine Laboratory, UK). RES = residuals.

| **Variable** | **Factor** | **Source** | **df** | **MS** | **Pseudo-F** | **P(perm)** |
| --- | --- | --- | --- | --- | --- | --- |
| Organic-C |  | MI | 1 | 12.18 | 94.93 | <0.001 |
|  |  | PL | 21 | 149.13 | 55.36 | <0.001 |
|  |  | MI x PL | 21 | 7.58 | 2.81 | <0.001 |
|  |  |  |  |  |  |  |
| MI x PL | Frissate | MI | 1 | 0.1 | 1.02 | 0.35 |
|  |  |  | 8 | 0.78 |  |  |
|  | Guenfouda | MI | 1 | 0.61 | 6.7 | 0.04 |
|  |  |  | 8 | 1.24 |  |  |
|  | Guercif | MI | 1 | 0.89 | 47.72 | <0.001 |
|  |  |  | 8 | 1.04 |  |  |
|  | Mezguitem2 | MI | 1 | 0.19 | 12.55 | 0.17 |
|  |  |  | 8 | 0.26 |  |  |
|  | Ogda | MI | 1 | 0.27 | 1.45 | 0.26 |
|  |  |  | 8 | 1.76 |  |  |
|  | Sabbab1 | MI | 1 | 1.12 | 9.23 | 0.02 |
|  |  |  | 8 | 2.1 |  |  |
|  | Sabbab2 | MI | 1 | 0.14 | 8.79 | 0.03 |
|  |  |  | 8 | 0.24 |  |  |
|  | Sahibat | MI | 1 | 0.74 | 2.72 | 0.14 |
|  |  |  | 8 | 0.29 |  |  |
|  | Saka1 | MI | 1 | 2.21 | 19.18 | <0.01 |
|  |  |  | 8 | 3.14 |  |  |
|  | Saka2 | MI | 1 | 1.93 | 7.8 | 0.03 |
|  |  |  | 8 | 3.39 |  |  |
|  | Barrax | MI | 1 | 0.99 | 0.36 | 0.62 |
|  |  |  | 8 | 2.68 |  |  |
|  | Huelves | MI | 1 | 3.74 | 16.01 | <0.01 |
|  |  |  | 8 | 5.14 |  |  |
|  | Morata | MI | 1 | 1.95 | 24.3 | <0.01 |
|  |  |  | 8 | 2.51 |  |  |
|  | Ontígola | MI | 1 | 1.05 | 5.17 | 0.05 |
|  |  |  | 8 | 2.67 |  |  |
|  | Sierra Espuña | MI | 1 | 0.75 | 7.5 | 0.02 |
|  |  |  | 8 | 1.43 |  |  |
|  | Yecla | MI | 1 | 2.11 | 5.11 | 0.05 |
|  |  |  | 8 | 4.99 |  |  |
|  | Zorita | MI | 1 | 0.85 | 4.65 | 0.68 |
|  |  |  | 8 | 2.14 |  |  |
|  | Bouhedma | MI | 1 | 1.14 | 98.8 | <0.01 |
|  |  |  | 8 | 1.24 |  |  |
|  | Matmata | MI | 1 | 0.24 | 17.69 | <0.01 |
|  |  |  | 8 | 0.35 |  |  |
|  | Sbeitla | MI | 1 | 0.59 | 0.24 | 0.64 |
|  |  |  | 8 | 2.06 |  |  |
|  | Sidi Bousid | MI | 1 | 0.04 | 0.17 | 0.7 |
|  |  |  | 8 | 0.17 |  |  |
|  | Tataouine | MI | 1 | 0.23 | 45.35 | <0.01 |
|  |  |  | 8 | 0.26 |  |  |

References:

1. Anderson MJ (2001) A new method for non-parametric multivariate analysis of variance. Austral Ecology 26: 32-46.
